# Ninth International Dental Congress, Washington, D. C., Sept., 1887

**Published:** 1887-11

**Authors:** 


### PROCEEDINGS.

### Ninth International Medical Congress, Washington, D. C., September, 1887.

SECTION XVIII. DENTAL AND ORAL SURGERY.

(Reported by Mrs. M. W. J. for Dental Register.)

The sessions of Section XVIII were held in the Universalist Church, corner of Thirteenth and L streets.

The section was called to order in first working session at 3.30 P. M., Monday, September 5, by Prof. J. Taft, Cincinnati, President of the Section.

Calling the members to order, and declaring the section formally opened, Dr. Taft called upon Dr. W. W. H. Thackston of Virginia, whose degree of D.D.S., he said, "antedated that of any living man," to speak the words of welcome.

Dr. Thackston addressed himself especially he said to his brothers from across the water; those who had signalized their devotion to the claims of science, their love of their profession by braving the perils of the ocean—greeting them in the name of the American members of the Dental and Oral Section of the International Medical Congress bidding them a hearty welcome "to our roof-trees; to our hearthstones; to our firesides; to our hearts."

Turning to Dr. W. H. Morgan (Nashville, Tenn.) he begged him to say for him that which he failed to find words to express, so extreme were his emotions of gratification and pride.

Dr. Morgan said that as the earth looks to the east for light, so do we look in that direction for light in science; the stars first make their appearance on that side and then we look for them on this. He was glad to take these foreign brethren by the hand and welcome them to friendly arms and hearts. He hoped that when they left this goodly city—the capital of our confederacy of States—they would realize that they had been both pleased and benefited by their stay.

Dr. W. B. Macleod, Edinburg; Dr. Metnitz, Vienna; Dr. Grevers, Amsterdam, and Dr. Sjorberg, Stockholm, responded on behalf of the foreign members of the section.

The President, Dr. J. Taft, then delivered his opening address, taking for his topic "The Factors and Forces in the Deve op ment of Dentistry."

Briefly analyzed, this was a concise, succinct, and masterly exposition of the history of the birth and development of four great factors in dental development, viz: Its literature, standard and journalistic; its association work, National, State, and local; its educational system, college and university departments, and its legislative features—forces which were never in better condition than now for continued work in building up. Its literature demands and has attained a high standard of excellence; its association work was never more appreciated; its educational institutions have entered on an upward and onward career; its legislative acts are stringent in ruling out the incompetent and the empirical. As a profession we are united; with no *isms*, no *pathies*, no factions to divide or waste our strength. The address closed with a ringing appeal to push forward to the fulfillment of high behests—of expectations ahead for work to be done in this section where are gathered representative men of the profession from all parts of the world—urging that these expectations be realized to the utmost.

At the conclusion of the address the Dental Society of Minnesota presented the President with an emblematic gavel in the shape of a mammoth molar, of white cement set in black rubber with gold bands—the rubber emblematic of elasticity of thought; the gold, of purity of motives; the cement, of the ties that bind all the members of the profession together—the whole representing something that is in every body's mouth, and that we are pained to part with.

The President received the gavel with thanks, predicting that all would now go smoothly, and that if any one was out of order he would yield to the pronunciamentos of such a gavel.

Thanks were voted to Dr. R. Finley Hunt, of Washington, D. C., for his untiring efforts in securing such ample accommodations and to the School Commisioners and Board of Managers for their liberality in giving the free use of the commodious Franklin School Building for the use of the Clinics and Dental Exhibits; also to the President for his able and valuable address.

Dr. R. J. Porre, Cincinnati, Ohio, then read a paper entitled Chronic Pyæmia from Dental Origin (for which see October REGISTER, page 487).

The discussion of Dr. Porre's paper was opened by Dr. J. Frank Lydston, Chicago, after which the section adjourned till Tuesday, 11 A. M., when the discussion was continued at some length.

Dr. Walker (dentist) London, England, expressed his surprise that Dr. Porre should so frequently use the forceps. According to his observations a great many cases were cured by treatment without extraction; in fact it was the general rule in England. He was much pleased with the address read by the Secretary (Dr. Lydston's paper opening the discussion) ascribing as the cause, microbes and their agency as assigned by Mills, Underwood, and other English authors.

Dr. Rehwinkle said that all were familiar with the pathology as described, but, as Prof. Dawson had once said to him, the idea of ascribing such serious results to a mere dental lesion was an absurdity in the eyes of most physicians a few years ago. The dentist who would have propounded such a theory to a physician five years ago would have been considered a fool, but now there are many physicians who send such cases to the dentist. The paper opens a wide field for discussion, and in pyæmia, as in pyorrhea, the dentist cannot confine himself to mere local treatment.

Dr. Brackett (Newport) thought that more careful examination would reveal necrosed bone far more frequently than was supposed.

Dr. Younger (San Francisco) wished to suggest two remedies—nitric acid for the dissolution of necrosed bone, from its great affinity for lime salts, and corrosive sublimate, 2 to 4-1000, as a disinfectant.

Dr. Genese (Baltimore) thought there was possibility of harm from the caustic and escharotic action of the remedies proposed, and preferred dilute aromatic sulphuric acid as safer, defining the line of demarcation between living and dead bone without fear of bad effects. He said that a small pellet of the solid extract of white poppies applied to the affected part would afford relief; it could also be dissolved in water and applied externally as a wash.

Dr. Whitefield advised electrolytic action for the destruction of microbes, using a platinum needle, with the other pole on the gum.

Dr. Storey (Dallas, Texas) asked whether the pus is supposed to enter the blood by direct absorption or was it taken up through the stomach and digestive process?

Dr. Chance (Portland, Oregon) showed a specimen of necrosed bone, from a case in which the trouble arose from a left lateral incisor, with caries of the maxillary bone, almost reaching the palatal bones.

Dr. W. C. Barrett (Buffalo) thought the discussion not up to the standard of medical science expected from the section. He objected to the term "pyogenic membrane," defining what was so called as a "scab" or cast of the cavity.

Dr. Porre, in closing the discussion, replied to Dr. Storey that the mode in which the pus was taken into the blood was left for the section to decide. It would vitiate the tissues in either or in both ways, or if the individual had sufficient strength and vitality he might throw it off and resist its effects for an indefinite time, perhaps for years, but he must eventually go under unless the cause were removed.

To Dr. Walker he replied that extraction was resorted to only when the tooth was past salvation. In all other cases he was opposed to extraction.

On motion the subject was passed.

The secretary next read a translation of a paper from Dr. E. Brasseur, Paris, president of the Odontological Society of France, entitled "De l'Air en Therapeutique Dentaire"—The Use of Air in Dental Therapeutics.

This was an exposition of the value to the dentist of hot air and medicinal vapors, applied by means of the thermo-injector.

He described the extremely sensitive points of dentine sometimes met with as "true ganglionic centers," and the teeth as

"perfect organs of feeling." He portrayed the evils following the use of arsenic for allaying the pain in sensitive dentine, also of carbolic acid, advising the use of vapor of carbolic acid, formed by heating the crystals in the canula of the thermo-injector, the vapor penetrating the canaliculi, and producing an anæsthetic instead of a caustic action. When dentine is discolored and partially softened, with the pulp nearly exposed, he employs the thermo-injector for applying heated air to exhaust the moisture of the dentine. Dressing immediately with bi-chloride of mercury, followed by oil of cloves and eugenol, he again injects heated air, and varnishes the cavity with copal varnish and paraffin, equal parts. Placing an asbestos wafer, soaked in oil of cloves, in the bottom of the cavity, he fills with oxyphosphate of zinc. When a pulp is first exposed, either by caries or accidentally, the pulp is but slightly sensitive. As disease progresses it becomes sensitive first to cold, then to heat and cold both, and in the last stages to heat only, this furnishing a key to the condition of the pulp. In the first stage every effort should be made to preserve the pulp alive, instructing the patient as to the importance of preserving the vitality of the tooth, that when dead it has no force to struggle against subsequent attacks of disease, that it is only held in place "by the ligaments of the pericemental membrane, that nature abhors dead matter and will make every effort to get rid of it, etc. If caries has penetrated to the pulp chamber it must be kept free from moisture and septic agents, which are certain to follow the presence of saliva in the cavity. Applying the rubber dam, the cavity is to be dried with spunk, followed by hot air, which will drive the pulp back if hernia has occurred through afflux of blood. Moisture causes the pulp to swell, but under the influence of hot air the pulp may be seen "drawing back like an earthworm." If not practicable to use a disk of platinum to cap the pulp, an operation perfectly feasible but requiring great dexterity, Rosenthal's Pulpine, or oil of cloves and anhydrous oxide of zinc, made to a paste-like cream, may be applied directly, and the cavity filled with oxyphosphate of zinc, with an amalgam surface.

If the pulp has reached the stage which indicates devitalization, or if gangrene has already occurred, with the aid of heated air we avoid interminable dressings. Applying the rubber dam, the walls are to be dried with absolute alcohol and dressed with bi-chloride of mercury, or bi-iodine, which is stronger. Anæsthetic vapors of chloroform, ether, or sulphide of carbon, under pressure of hot air, are very penetrating and abstract the water which is normally present, a small quantity of vapor eliminating a comparatively large amount of water. These vapors "do not tarnish the brilliancy of the enamel, so precious in the front teeth." The use of these vapors and those of carbolic acid and iodoform, by means of the thermo-injector, will transfer a large number of cases called incurables to the ranks of conservatism, and materially contract the limits of extraction.

As long as any putrefactive elements remain pus-formation will continue. One or two drops of carbolic acid in the root canal, followed by soft gutta percha, will drive all pus in the canal through the apex to the fistulous opening, where bubbles will go out, showing that it has penetrated. Clearing the root canal and opening the apex with a root-trimmer, tannin and iodoform can be placed in the canula of the injector and blown through the apex by compressed hot air to the walls of the abscess cavity. Placing a wad of felt at the apex, fill with gutta percha. The cure is complete when the wad is odorless, when the permanent filling may be inserted. In chronic periodontitis a douche of hot air on the neck of the tooth penetrates to the bottom of the affected part and affords prompt relief.

The convenient application of hot air is valuable to the dentist in many other ways. It gives gutta percha greater plasticity, renders amalgam more plastic, is invaluable in drying the surface of gold fillings, which may be accidentally wet by saliva or the breath, and hastens the setting of cement fillings, which, when protected by paraffin or copal varnish, often enable teeth which were mere wrecks to do good service. It is also valuable for the application of bleaching agents, causing them to penetrate every portion of the discolored dentine; also for the application of medicated vapors in diseases of the antrum and in catarrh.

The hour for adjournment having arrived, the discussion of the paper was postponed till the afternoon session, at 3 P. M.

TUESDAY, 3 P. M.—The discussion of Dr. Brasseur's paper was opened by a written paper by Dr. C. A. Brackett, Newport, Rhode Island. Dr. Brackett said that having studied the paper in the original he was perhaps better able to judge of its value than those who had listened to a translation of portions of the paper, which as a whole was too long for the time allotted to it, and which for that reason scarcely did justice to the author. The prominent idea of the paper was that of lessening sensitiveness of dentine by hot air in the process of drying, eliminating the normally contained water of the tissues; in proportion as its moisture is diminished so is its capacity for either normal or pathological functions. When bone is burned in the fire it loses certain elements. If it could then be replaced in its position in the body it would not be capable of performing its normal functions. The same would be true if certain elements were dissolved out by acids. Whether dentine loses only one or more than one of its normal constituents it is that much less than dentine; it is imperfect and incapable of rightly performing its proper functions, one of which is that of conveying sensation. This function is performed by the living portion, not by the lime salts. The water of the tissue is an important element in its constituents, making the essential difference between the living organ and the dead, dry, extracted tooth. If this is eliminated from the tooth while in position in the mouth it becomes less capable of performing its normal function of conveying sensation. It can, however, without injury be eliminated from the walls of a cavity which does not approach the pulp. In cleansing a cavity of decay the mechanical work is now supplemented by the introduction of antiseptic agents. The use of arsenious acid for sensitive dentine is never justifiable. The author of the paper recognizes the different pathological conditions of the pulp. His views of pulp-capping are not new to us. Hygrometric filling material is not always objectionable; it is sometimes both comfortable and successful, even if it absorbs the slight exudation from the pulp surface. Great credit is due to the author for the apparatus invented, though the advantage of blowing in dry medicinal powders, over liquids conveyed by the syringe, is questionable. He speaks of air heated to 40° or 45°, but of course this is not Fahrenheit. The term "dead teeth" was used by the translator when pulpless teeth with the pericemental connection intact were spoken of by the author.

(Dr. Dudley here passed around the illustrations accompanying the paper, explaining that the apparatus was of two forms constructed so as to have the heat generated either by gas or petroleum, or electricity.)

Dr. Truman (Philadelphia) said that in the use of hot air, as of any other agent it must be born in mind that the tooth is not a dead body; the pulp has its prolongation through the dentine, which, though microscopic, is no less sensitive than the pulp itself, so that if heat is carried past a certain degree, it might destroy those delicate ramifications, or convey such irritation to the pulp as to cause its death. Great caution should be used in the experimental use of any new agent.

Dr. W. H. Morgan (Nashville) thought the statements in the paper too broad, and the language not sufficiently exact. The proposition was simply to dry the tooth with heated atmospheric air; but the atmosphere might be so saturated with moisture that it would not dry at all? Again, he said "we are justified in treating pulpless teeth with the strongest antiseptics known to surgery"—first pump in hot air till the tooth is dessicated, and then refill all the spaces with fluid antiseptics; I commend that view to your attention. Another broad assertion was that absolute dryness is essential to the success of a gold filling! There are many gray-heads among us who put in gold fillings thirty or forty years ago, before the day of rubber-dam, who know that that is not exactly true. We made many submarine fillings that did good service. The cavity may be moist with alcohol, or chloroform, or carbolic acid, and not harm the filling. He took broad ground also in his description of the *treatment* of alveolar abscess; he made no exceptions. The old fundamental principle of medicine—remove the cause, and nature will effect the cure, is as true in alveolar abscess as anywhere else. If we remove the cause and arrest putrefaction and decomposition, nature will do the rest in a large majority of cases, without any *treatment* at our hands.

Dr. Geo. Whitefield (Evanston, Ill.) said that the surest way to avoid pain in excavating sensitive dentine was to use a very rapidly revolving keen burr, which would not clog and produce friction; the first instant of pain would be all.

The decomposition of common salt with electricity would liberate chlorine and bleach discolored teeth in twenty-five minutes. The brine would penetrate dry dentine and liberate the chlorine in the tubuli, whitening the dentine very rapidly.

Dr. Guilford (Philadelphia) said that the use of hot air for reducing the pain of operating on sensitive dentine promised to be a valuable adjunct. If all moisture could be evaporated by any artificial means, we would get rid of all sensitiveness. We see this when two teeth are isolated by the rubber-dam. While we operate upon the first the second gets comparatively dry, and there is but little sensitiveness. Dr. Guilford described in detail the construction of an apparatus for the storage of compressed air for one at the chair, either hot for drying and dessicating live teeth, or cold for devitalized teeth, at will. For setting pivot or dowel teeth the roots can be so thoroughly dessicated as to have the appearance of chalk, taking a long time to get moist again. The effect of hot air on sensitive live teeth is surprising to one who has never tried it, and very satisfactory both to patient and operator. The pressure can also be used to spray medicaments, but the satisfactory results in excavating sensitive dentine, and in setting dowel teeth will fully repay the cost and trouble of getting the apparatus in place.

Dr. Brockway (Brooklyn) thought such elaborate apparatus frightfully cumbersome, and absolutely needless, the chip syringe and warm air from the flame of the alcohol lamp accomplishing the same results, with no outlay beyond the help of an assistant, which is just as necessary for a successful and easy practice as are the burrs, engine, etc. He had tried the dessicating system for some time, but had abandoned it for the reason given by Dr. Truman—the danger of injury from excessive dryness. He now overcame sensitiveness by using burrs sharply cut and kept wet with a stream of tepid water. He found this perfectly satisfactory.

The time allowed for the discussion of this paper having elapsed,

Dr. J. Cravens (Indianapolis) read a paper entitled "The Management of Pulpless Teeth."

Dr. Cravens said that a longer tenure of usefulness could be insured pulpless teeth if they could be treated without the injection of medicines through the canal, which saturate the dentine and are injurious to the pericemental membrane. A pulpless tooth is not dead. The cementum maintains a vital connection between the dentine and the pericemental membrane, affording collateral sustenance and preserving the tooth in apparent health, comfort, and usefulness for many years. If this function of the pericementum is impaired or perverted then the pulpless tooth dies and becomes offensive to the economy. The apical space—a dense, dark crypt—is invaded by a bristle through the nerve canal, letting in light to what then becomes a battlefield—a *champs de Mars* for hobby-horses with their riders astride. The idea seems to prevail that the root canal was designed solely for the introdnction of remedies, but the very nature of the parts suggests that it is impracticable to accommodate much medicine. The pericementum is closely confined between the cementum and the walls of the alveolus, with barely room for the membrane in health. If inflamed it thickens and needs more space. To make more room for itself it lifts the root in the socket, and the elevated crown becomes painful in occlusion. If we force medicines in where there is no room we must have pericemental inflammation, and alveolar abscess to follow. Recently a few are eschewing irritants and escharotics. The change is an improvement and a half confession of the folly of their indiscriminate use. Medicines are applied to the cavity of decay, and not allowed, or designed, to go beyond. But why? If the objective point is the congested membrane the remedies must go through the apical foramen to reach it; but the inflammation is sometimes quite remote from the foramen. In molars it is usually confined to the crotch at the junction of the roots. When the tooth is painful under percussion we know the pericemental membrane is inflamed, but we don't know about which root to locate the tract; decay does not locate it. The theory of blind abscess is all guess-work. It is a convenient diagnosis for flooding the canal with medicines which often create an abscess, when the simple application of oil of cloves would often avert all trouble, and not be followed by any inflammation. With a large number of practitioners pulp canals must always be *treated* before filling. Medicines are injected, the tubuli absorb the fluids, and by capillary attraction they are carried to the periphery of the root; the cementum is invaded and the pericementum is reached, and the medicines work down along the outside of the roots. In a few weeks, or perhaps years, the effects are seen in an irritating exudation through a fistulous opening, or at the gingival borders. The individual may detect the medicine which was placed in the pulp canal years previously. Finally the pericementum is hypertrophied, its function as conservator of the cementum is perverted, and the tooth is cast off as a dead body. The methods of this paper require that the apical end of the canal be closed as soon as free from pus or other fluids and obstructive matter. Clean the canal till laudable; if the apex is properly and promptly closed, no matter what is used for filling the canal; medicine is of no assistance to manipulation. Broken down pulp tissue should be removed by barbed broaches, gaseous contents by mechanical displacement; absorbent cotton on a broach making a swab small enough to pass entirely through the canal, avoiding compression and allowing the gasses to pass by and out, pure air will go in to fill the space vacated by the gas to satisfy the vacuum. This simple process will effectually cleanse a root canal of sulphuretted hydrogen and other gas. The fluid contents will be absorbed by the cotton of the swab, which should be frequently changed for fresh, pressure being carefully avoided, that nothing be forced through into the apical space. If the swab fits too closely the contents of the canal will be compressed and forced through, and inflammation and abscess created. An easy and safe way of closing the apex is to cut No. 10 tinfoil into narrow strips and carry one end singly and slowly to the end of the canal, compacting it by gentle manipulation with a springy canal plugger. This method recognizes the sanctity which hedges the apical space. Whatever enters there is an impudent intruder, and we get a prompt expression of the indignation of the offended membrane. An abscess with fistulous opening is a complication.

If pus is formed by the disturbance it should discharge through the pulp canal; that is its natural exit. It can be cleaned away by the operator, as described, till the canal is laudable, when the apex may be permanently closed. If it forms a fistulous opening the fistula will accomplish the drainage of pus from the abscess; it will heal when no more pus is formed, usually in a few weeks, without canal medication. If the tooth is too sore to allow of opening the pulp cavity, it is better to wait for a decline of the soreness than to force conclusions; in the end this will prove more expeditious. A fistula is the dentist's friend and best ally for drainage, relieving the canal. Encourage them to perform their good office without interference; they will disappear when their good work is done. For deciduous teeth the method is much the same, but fill them with phosphate of lime, in the magma state. The water is removed by the absorbents. But do not fill close to the end of the root in deciduous teeth. Deciduous teeth with fistulas in the gum surrender to this method in one or two days; but do not use any medicine in the pulp canal of deciduous teeth, and force is inadmissable. In many adult teeth the pulp may be removed and the tooth filled at once without any medication. The roots of deciduous teeth are resorbed after they have been filled with phosphate of lime, but we have no means of knowing how much had been resorbed before filling.

While admitting that these methods are at right angles to the tenets of the profession, Dr. Cravens hoped they might be given fair trial, and if found as satisfactory by others as they had been by himself we could say, "throw physic to the dogs."

The opening paper of the discussion was read by Thomas Fillebrown, M.D., D.D.S., Portland, Maine.

He said that while it was strictly true that cleanliness and dryness were essential points in treatment, it was not so clear to him that medicine was always harmful. Over medication is no doubt harmful, and non-interference might be better in some cases, but it is difficult to say what might have been or what nature would have done. Great and uniform success is claimed by many for non-interference; but there are different standards of success. In case of chronic abscess, with no fistula, he would not risk closing the apex at once. He would consider the inflammation outside of the tooth a persistent secondary cause, and that closing up the canal would not cause healing. He could not expect to stop the discharge by closing the opening for discharge. There would be danger of pyæmia, which he would not have the temerity to risk. Possibly a filling which was porous, absorbing the discharge, might be tolerated, because nature sometimes tolerates a large amount of poison. He thought when the tooth was "too sore to open" the true remedy was to open at once with sharp instruments, saving days of agony by a few moments pain, avoiding suppuration by allowing the pent-up contents to escape. The "apical space," spoken of in Dr. Cravens' paper, was to him a myth. The cortical substance of the alveolar wall closely invests the root of the tooth, the membrane being closely adherent, with no space unless excavated by art, or through absorption by disease. Hypertrophy, discoloration, etc., were more frequently the results of retained pulp than of medicines. If the pulp was removed discoloration would be very slow. If there was blood-coloring in the tubuli discoloration would follow in spite of medication or non-medication. It was not fair to charge to medication what was due to percolation or osmosis. If root canals were filled with cotton and drugs, and fluids forced through the foramen, then great injury was liable to ensue, and possibly loss of the organ. The treatment of deciduous teeth laid down by Dr. Cravens was not consistent. He says, "use no medicines in deciduous teeth," and then proceeds to fill them with phosphate of lime—an antacid and insoluble. What becomes of it when the roots are resorbed? Most operators leave such roots unfilled. If this method is a success it is worthy of consideration and trial. Dr. Fillebrown said that for twenty years his method had been to treat abscess through the fistula, and not through the canal, packing the canal with cotton and sandarac to test it until the inflammation of the tissues was reduced by sedatives. Dr. Craven's "system" could not be called "complete," as he failed to consider numerous other conditions, as when the end of the canal could not be reached, etc.

Dr. W. C. Barrett, Buffalo, New York, criticized the paper quite severely, though according to it but little significance. He said that its pathology was more than singular, its etymology peculiar, its orthography *sui-generis.* He did not consider such pathology, or rather such lack of pathological knowledge worthy of serious consideration by the International Medical Congress. The subject was viewed from the standpoint of twenty-five years ago. In the past five years great advances have been made that we cannot be expected to ignore in a meeting of this kind, to go back to the discussion of methods exploded twenty years ago. It was not up to the requirements of the occasion, and should not go forth to the world as marking the status of American dentistry. It is a duty we owe to ourselves and to our profession, to our microscopists to recognize the results of their labors for the past ten or fifteen years in establishing the facts of the septic condition of such teeth and the necessity for antiseptic medication. To set forth the principles of antiseptic treatment of the present day; the first thing to be done under all circumstances is to give egress to septic matters; then proper remedies must be introduced to disinfect the territory; then germ-destroying agents to prevent the reproduction of such products. These are the three steps always necessary in the conservation of teeth with dead pulps.

Dr. A. W. Harlan, Chicago, said that though the title of the paper was a "System for the Management of Pulpless Teeth," it read like a medical romance. Dealing with modern advances in antiseptic surgery and bacteriology, the author of the paper says he banishes mephitic germs by swabbing the canals whence pus is flowing, rendering canals "laudable" in that manner, forgetting that the entire dentine becomes saturated and polluted with those gasses. The paper is too far behind the age to go forth from this Medical Congress as the opinions of the advanced American dentists of to-day. It is *absurd* to say the least to suggest that we discard all antiseptic and disinfectant treatment, and go back to the mere mechanical methods of forty years ago. The author seems to think that the dead pulp produces no injurious effects, but that the object of medication is to cure some disease of the canal itself. If odors could be mechanically displaced (which we deny) ; if we stop the flow from the tooth—which means the drainage of the sac beyond, and fill without disinfecting, disaster will follow unless it finds a fistulous outlet. The gentleman says in his remarkable paper that it is not certain that the root canal contains pus ! It is true the eye will not detect it, but any man with reputable knowledge of microscopy will detect it at once. The paper assumes that it is the practice of the American dentist of to-day to force escharotics into and beyond the apex when there is no fistula. Is it true that it is customary to inject escharotics into a blind abscess with no outlet ? We know that it would inevitably produce disaster.

This " System " as a system is unworthy of our consideration ; unworthy of our knowledge of microbian diseases.

Dr. W. H. Morgan said that the gentleman who had just taken his seat was mistaken in stating that any such *system* had been in practice within twenty or twenty-five years. If he would go back and read papers published in the journals of 1854 or 1855 he would find that they advocated every therapeutic principle adopted to-day. It may be true they did not have many of the remedies used to-day, but the therapeutic principles in-involved were the same, and were as well understood. We have been told not to fill the roots of deciduous teeth *because* the roots will be resorbed and the metal left projecting ; that is—we would have physiological action in a dead body ! It is true the entire structure is not devitalized because the pulp is dead, but the dentine is dead and the physiological process of absorption is arrested except in the cementum. A dead tooth may be broken down by chemical action, but not by a physiological process. The only remedy for pulpless deciduous teeth is extraction.

Dr. Butler (Cleveland, Ohio,) said that the present paper and its discussion reminded him of the debating societies where men took opposite sides in order to bring out all the points. He could not conceive that any one posted on pathological principles could project such a " *system* " without having a similar object in view. It had certainly brought out some earnest and intelligent advocates of the other side, and he hoped our friends from the other side of the Atlantic would understand it. Certainly the results seen in this discussion could not have been reached in any other way, and we should therefore take a charitable view of the paper because of the good results it had brought about.

Dr. Harding (Shrewsbury, England,) said that since he had been in America he had seen much to excite his wonder and admiration, but nothing had astonished him quite as much as the paper just read. In England they were imbued with the ideas promulgated by Prof. Lister, based on the researches of Tyndall and others, on the subject of pus and septic matter. To be gravely told by the writer of the paper that septic products can be gotten rid of by mechanical treatment excites both interest and astonishment. He must think, as one gentleman has suggested, that it was possibly written simply to elicit discussion, and was glad to see that it was not the general opinion that septic conditions could be cured by mechanical treatment. When pus is formed from a dead pulp it is certain to infiltrate all the structures around, and as long as the germs have pabulum to feed upon they will be reproduced. The theory that germicides applied to the interior of the canal will produce inflammation of the periosteum is absolutely unfounded, provided, of course, that the remedies are not forced through the apex. When there are germs, an exit must be provided; they must not be plugged up. When the last germ has been devitalized then we can fill with success.

Dr. Cunningham (Cambridge, England,) had hoped to have the privilege of laying before the section statistics of over five hundred cases filled by the "immediate" method, which might induce them to look more leniently upon the essayist of the afternoon. Unfortunately his effects were not at hand, but he would ask permission to produce his statistics towards the close of the meeting if his papers arrived in time. He thought that his diagrams and figures would add interest to the present discussion.

Dr. Craven said that the discussion had been altogether on one side, he would postpone his own concluding remarks in order to give the gentleman who had just spoken, opportunity to present his statistics.

Discussion closed for the time being.

Dr. T. E. Weeks (Minneapolis) then read a paper entitled :

### Matrices as Adjuncts in Filling Teeth.

He said that the object of matrices was to convert compound into simple cavities having four walls. They were especially useful in filling approximal cavities of molars and bicuspids. It was impossible for any one shape or form to be used to the exclusion of all others, especially of the rigid kind. Almost any thing can be used as a matrix, as steel pens, clock spring stuff, pieces of broken separating files, etc. He then described a number of the different patented matrices in the market, as those of Drs. Louis Jack, J. A. Woodward, D. R. Jennings, of Cleveland, Ohio, W. B. Miller, H. A. Whitney, D. B. Freeman, E. T. Darby, S. H. Guilford, A. C. Hewitt, Latimer Brunson, Herbert, Drs. Henry, E. B. Cattle, Huett, Finney, Kreeger, Truman W. Brophy, and many others, illustrated by a series of charts, showing the application of a few principles, variously modified, in the use of flattened wire, strips and bands, entire circles and partial loops, held in place by wedges, solder, shellac, springs, set screws, clamp screws, bolt and nut, etc., the essential requisites being close adaptation to the tooth, adaptability to the greatest number of cases, non-corrosible material, pliability sufficient to admit of flush fillings at the margins of cavities, the least cumbersome being generally the best. It would seem, from the great variety in the patented matrices, that every emergency might be met, and yet every dentist should have the ingenuity to devise and the ability to make a matrix from material at hand, for cases will occur that no ready-made matrix will fit. We should not forget that our neighbor's methods may be quite as good as our own, though perhaps very different. We must guard against riding a hobby till it throws us into a ditch.

The opening of the discussion was assigned to Dr. S. H. Guilford (Philadelphia).

He said that it must be borne in mind that a matrix was an appliance intended to supply a lost wall, its object being to enable us to perform a difficult operation with greater facility. They were made of a great variety of materials and in great variety of forms.

It is unnecessary to argue in favor of the matrix as an adjunct, since the profession so generally admits their incalculable value. Nevertheless, there are men who argue against their use, who totally condemn them, who fail to see any advantage in their use. Their arguments against the matrix are that it is hard to absolutely fill the space, and for fear of not having enough an excess is used, which it is difficult to dress off; that the portion built against the matrix is unlike the original wall; that the artificial wall overlaps the natural wall, so that it is impossible to get a perfect margin; that the filling goes either beyond or not quite to the edge of the cavity, etc. But unless we have the skill of a Marshall Webb, to which few can hope to attain, it is much easier to reduce a compound filling to a simple one by restoring the lost wall by the aid of a matrix, doing the work with a minimum of gold, and saving time to both operator and patient. A matrix, to be properly constructed, should be only a little yielding, giving the least surplus to be removed, which is readily done with corundum tape. The great advantage is the saving of labor and time.

This paper and Dr. Guilford's reply covered the ground so completely, enumerating both the advantages of and the objections to the matrix, with arguments *pro* and *con*, that no room was left for discussion. The subject was accordingly passed after the reading of the opening paper by Dr. S. H. Guilford.

WEDNESDAY, Sept. 7, 11 A. M. The opening paper was from le Docteur Pradere de Moine, Lyons, France, and entitled "*La Phthisie raincue par la Permanence des Medicaments au Milieu du Palais*," or

PHTHISIS CURED BY THE CONTINUOUS APPLICATION OF MEDICINE TO THE PALATE.

The Secretary, Dr. Dudley, read a translation of portions of the paper, which, he said, was very long, and much of it of no special interest to dentists.

Dr. Pradere presented with the paper his appliance for the continuous inhalation of vapors, which he calls a "permanent invisible dental inhaler"—*dental* because it may be made by the mechanical dentist, and because it is worn in the roof of the mouth, like a plate for artificial teeth. It holds from two to four drachms of fluid or semi-fluid medicines, held by absorbent cotton in the appliance, and which may be changed every few hours and worn night and day, securing a permanent continuous inhalation of such vapors as kill the microbes of tuberculosis and other diseases. In ordinary modes of treatment, which are intermittent, when treatment ceases the ravages of disease are renewed, and the effects are only palliative, not curative; nothing is accomplished towards curing the poisoned internal wounds. In the ordinary modes of administration of remedies the inflamed mucous membrane of the stomach opposes the absorption of the medicines, and a chemical reaction takes place before the blood is reached, and the septic matters are not neutralized. By Dr. Pradere's method vapors are continuously inhaled into the lungs and swallowed with the saliva, so that all portions of the sytem are reached, even the microbes, which produce tubercles in the mesentery and soft portions of the connective tissues. The remedies used in the inhaler are of course varied to suit the case, employing carbolic acid, creosote, napthaline, menthol, etc. The medicine itself does not touch the tongue or the mucous membrane, but reaches the respiratory and digestive tracts being taken into the stomach with the saliva, proving of great advantage in dyspeptic troubles, etc. Poisons are rooted out, bacteriæ development is arrested, putrid fermentation ceases. The most remote traumatic portions, the deepest seated caries are reached as by no other known method of treatment. Numerous formulas were given by Dr. Pradere, and instances cited showing rapid improvement in very serious cases—deep seated cough being eased in from two to five days, expectoration ceasing in ten days. In a case of diabetes quoted, 50 drachms of glucose in 24 hours were reduced to 4 drachms after ten days' treatment. Benefit was suggested in case of soft and spongy gums, offensive breath, abscesses, and wherever anti-parasitic remedies were indicated.

The discussion of this paper was opened by Dr. A. W. Harlan (Chicago).

He did not think the paper worthy the attention of the section, and its indorsement was not probable. The name of the author does not appear in medical literature, and it is apparently an advertisement rather than a scientific paper. It is based on false principles. Violent remedies of which the ordinary dose is perhaps three minims are prescribed in doses of from two to four drachms every fonr hours. With such treatment the patient is not likely to need a second dose of this permanent invisible medication. On the other hand, the vapors of creosote, etc., are insufficient and inadequate for the destruction of the bacillus tuburculosis.

The essence of mint plays an unimportant part which it is not necessary to consider. Napthaline is insoluble in water—to use it in this permanent invisible inhaler it would have to be first dissolved in alcohol or some essential oil, and there would be no vapor to reach the cavities—it is therefore utterly useless. As a therapeutic agent camphor will not conquer tuburculosis, while in the prescribed doses it would soon produce trouble in the gastric regions. Its vapors are inert for bacillus tuburculosis. In short, the benefits to be derived from this apparatus and its absorbent cotton are simply *not proven.*

Dr. J. S. Marshall (Chicago) thought the author of the paper had made a mistake in presenting his paper to the dental section. It should have gone to the section of Practical Medicine, and in the second place the Committee on Papers had made a mistake in not sending it where it belonged. He suggested that our specialty was not competent to discuss the paper. There were but two points in the paper of any possible interest to the dental profession; one was the mechanical construction; the other the possible value of the treatment in oral pathological conditions. As the reader had omitted his formulas there was nothing to be said on that head.

Dr. Frank Abbott (New York) said that the paper had been submitted to him, and he had written to Dr. Dudley that in his judgment it should not be brought before this section. Except in the mechanical construction of the appliance it belonged entirely to practical medicine.

Dr. Truman (Philadelphia) could not commend the action of the committee in bringing the paper before this section.

Dr. Abbott agreed with Dr. Truman and moved that it be struck from the list of papers of Section XVIII.

Dr. Marshall offered to amend by referring it to the Section on Practical Medicine.

The President, Dr. Taft, thought the indications given were sufficient to demand action on the part of the section, but thought a direct vote unnecessary. It would be sufficient for the Secretary to refer the paper to the proper section. They had, of course, a perfect right to express an opinion, but it was not always advisable.

Dr. Truman, while appreciating the motives of the President, thought it no reflection on the paper or its author to refer it to another section, and thought the President bound to put the motion which had been duly seconded.

The President, deferring to the voice of the section, referred the paper to the other section.

Dr. J. V. Metnitz (Vienna, Austria) then read a paper entitled

### OSTEO-MYELITIS.

The paper was read partly in the original German, and partly translated at sight. He said that inflammation of the medullary substance of the maxillaries was seldom met with outside of the hospitals, but there, cases of complete necrosis and death were often encountered, probably aggravated by uncleanliness on the part of the patient, and perhaps by unclean instruments also. The original causes were hidden and not easily discovered. In the case recorded, of which photographs were exhibited, the patient was forty-three years of age when a number of teeth were extracted. He was soon after taken with high fever; on the fifth day he was delirious, and on the seventh day was taken to the hospital. The stench was then intolerable and his left cheek and temporal region immensely swollen; the eye ball distended, and the jaws set so that the mouth could not be opened; the muscles of the neck were also stiff. On the following day the patient died. Examination of the oral cavity showed both upper and lower left alveoli empty except the remains of the third molar which came away in shreds leaving a socket six millimetres deep. The articulation of the left side was completely destroyed and there was necrosed bone in all directions.

In a second case, a patient of seventeen years old, there was chronic inflammation of the lower maxillary. The periosteum was destroyed and there was no nutrition of the parts—removed a sequestrum fifty millimetres long of spongy bone. The direct cause of death was meningitis.

Dr. M. L. Rhein (New York) opened the discussion (without the usual written paper). He said the paper was of much interest. The author appeared to think it advisable not to incur the risks of interference, but if he understood him correctly he must differ as the free use of the burr in removing all infiltrated bone would often produce beneficial results, if we went beyond the line of infiltration.

Dr. G. J. Fredrichs (New Orleans) said the treatment of the author amounted to nothing because the patient died. He only gave the history of the cases. He himself had had no experience in cases causing death, though it might sometimes be necessary to excise the entire lower maxillary to effect a cure.

The subject passed without further discussion.

Dr. M. G. Jeninson (Minneapolis, Minnesota), read a paper on

ART IN DENTISTRY.

He said that the perfection of talent lay in the application of the principles of art to practice, for which dentistry opened the widest field. That there were so many lamentable failures lay in the fact that money was made the principle object. Colleges and preceptors are responsible for allowing men to enter the profession who think that it is simply an easy way to make a living, but who have no appreciation of beauty or expression. In true dentistry there is an opportunity for art at every step. In restoring the teeth to usefulness and beauty it must be born in mind that there was a definite purpose in every line, in every form, in every surface ; to cut away anything is to destroy that purpose. A very slight loss will change both articulation and expression of features. In prosthetic dentistry especially, the greatest opportunities are offered, for health and happiness are both at stake.

With diseased teeth the secretions are vitiated and the foundation is laid for dyspepsia and all its chain of troubles. In the right hands an edentulous patient can be restored, and sometimes even nature improved upon, but this is not the work of Cheap John or of incompetent assistants. The profile of Apollo is the type of physical beauty, but the slightest modification of any feature would detract materially from that beauty—"if the nose be shortened one-sixteenth of an inch the god is destroyed." The face of a patient must be made the subject of careful study, especially when he is not aware of it. If the bite is shortened, if the depression above the chin is filled out, if the lips are allowed to droop, the whole expression is changed. The play of the muscles of the mouth must be watched, when open, when closed, when in full action, when in repose. What is pleasing in one would be a deformity in another. In the arrangement of the teeth there are great opportunities for restoration and beautifying. The teeth too should be selected with reference to both temperament and age. Teeth as they come to us from the manufacturers, in gum sections, answer very well for a "pretty set," but they afford no scope for originality.

The discussion of this paper was opened by Dr. John Allen (New York).

Dr. Allen spoke of the antiquity of dental operations as shown in the tombs of prehistoric races, in Egyptian mummies, and in the works of Greek and Latin poets, and medical authors from Galen down, but it is only in the present century that elaborate works on dentistry have been published, and that we have dental journals, dental colleges, and dental associations, with harmony and unanimity among the members, all working to promote the best interests of the profession. With these facilities dental science stands higher than ever before; yet there are greater heights to be attained, especially in the artificial department. It is not *mechanical* work, for there are no rules, no written laws to govern as in pure mechanics. There are no two cases alike; there must be practical perception to meet the individual requirements of each case. The restoration of form and feature and expression to mouth and face is not mechanical work, it is art, and requires the highest degree of artistic skill; far more than mere canvas work. The human countenance expresses joy and love and sorrow and gratitude. "The heart of man changes his countenance." We must note what changes take place in the face of a patient, in his changes of mood, and devise ways and means to meet the requirements of each case. A certain degree of knowledge can be acquired through books, but that is soon exhausted, and for the higher practical points we must depend on our powers of conception, our own individual powers. Such individual efforts are the means of advancing dental science. Our ideas may be at first crude and chaotic, but they should not be allowed to perish for want of nourishment; they should be nurtured and cherished—"Wait till the little dawnings awake with full clear light." The knowledge that is conquered by labor is truly our own. Through such individual efforts our profession will advance, nourished by the dew of wisdom, warmed by the sun of science.

The subject was passed without further discussion.

The session of Wednesday afternoon was held in the National theatre, which was darkened to allow of stereopticon illustrations of the papers read, and an exhibit of photo-micrographs.

(The auditorium being dark notes of the papers read were taken under difficulties.)

The first paper read was by Dr. R. R. Andrews (Cambridge, Massachusetts,) on

### The Origin of the Dental Fibril.

Different processes have been adopted and different methods used, by different investigators in their efforts to get at the exact structure and minute details of the process of calcification and origin of the dental fibril. When it is possible to work nearer life, we may hope to reach more satisfactory results. The methods used in obtaining the specimens from which the present illustrations were made, are somewhat different from those of late writers. The calcarious tissue is taken from the teeth of the embryo, at about the time for birth, and placed in from one-quarter to one per cent. chromic acid, which is changed daily for two or three days. The edges of the dentine will be softened and a number of sections can be made. The material is usually placed in alcohol to take the water out of the tissues, but if paraffine or lard is melted in a mold, and when clouded, the tissue—dried on bibulous paper—is placed in it and left to cool, very thin sections can be cut; each cut turns the edge of the microtome, but we work as near life as is possible with our present knowledge.

The text of the paper requires the illustrations which were thrown upon the screen—photo-micrographs which were actual representations of tissue, the author guaranteeing that not a single line had been added to carry out a theory, all the work having been done by himself from the selection of the material and cutting of sections to mounting the photo-slides. The first was a section from the jaw of an embryo pig, two inches in length, showing the first dipping down of a line of epithelium, a minute tubular gland in appearance.

The next was an enlarged view of the same tissue showing to better advantage.

In the third illustration cells were forming in the lower portion of the gland-like body giving it a flask shape.

In the next the cells were enlarged and still further changed in shape.

In the fifth the borders are changing and the dentine growing up in the form of a papilla.

The next illustration shows the papilla below the enamel caps.

The next shows a section of a sheep's jaw with the enamel organ and papilla larger than in the preceding.

In the next the papilla has assumed the shape of a tooth with the permanent tooth germ starting.

In the breaking up of tissues this sometimes wanders off and is absorbed, or becomes a supernumerary tooth.

In the eighth was shown the point of the growing tooth, with connective tissue elements, the pulp clinging to the wall of the dental sacculus and the cement organ. Budding from the side of the enamel organ, is the bud for the permanent tooth, which goes through the same stages as the deciduous tooth.

The next illustration shows the beginning of calcification, with the odontoblasts and fibrils forming.

In the next the layer of odontoblasts and fibril cells was more plainly shown, with formed enamel; also the permanent tooth below.

The eleventh and twelfth were in strong contrast, the first showing the odontoblasts and membranous protoplasm as shown by the old methods; the other defining clearly the pear-shaped fibril cells and the fibril itself drawn out from one of the broken cells. In succeeding illustrations the odontoblasts were shown to be square and abrupt edged, the fibril cells being elongated and pear-shaped, with distinct line of demarcation, the fibrils seen passing from the pulp between the odontoblasts to the pear-shaped cells.

The last but one of the views shows the mature pulp against the dentine.

In the last was seen a section of the entire jaw bone and all the tissues; the dentine and enamel with the wavy lines anastomosing between the two layers.

(This report, partly from memory and partly from notes taken in the dark, necessarily fails to do any thing like justice to the language of the speaker in pointing out the leading features of the magnificent series of illustrations projected upon the screen in rapid succession.—*Reporter.*)

Dr. Frank Abbott (New York), in opening the discussion, said that he hardly knew what to say—the illustrations so far exceeded any thing he had anticipated. The profession was deeply indebted to Dr. Andrews for the time and labor bestowed for the education of the dental profession. And yet, as he (Abbott) understands the subject, the dentil fibril does not originate as Prof. Andrews puts it. Not having the material at hand, he was not able to demonstrate his views. He was sorry Dr. Andrews had not said more about calcification. Fibrils issue from any part of the walls of the odontoblasts—from end or sides, some becoming pear-shaped or spindle-shaped from being compressed and crowded in—some being square, some spindle-shaped, some wedge-shaped, and other awkward shapes due to their peculiar position. When the first row receives lime salts and becomes formed dentine a second row is formed behind the first, projections passing up the sides, between the first; all the living tissue in the dentine starting from the pulp, passing between and into the odontoblasts and spindle-shaped cells. Lime salts are deposited around all the cells, the fibrils being preserved as living tissue.

The second paper, also illustrated by the projecting microscope, was by Dr. M. H. Fletcher (Cincinnati,) on

### Protective Dentine or Dentine of Repair.

The so-called secondary dentine which the pulp throws out is for its protection from external injury or from exposure. In this protective dentine there is great irregularity in the dentinal tubes, with a larger percent of basis substance than in normal dentine; it is more translucent than is normal. This also includes the unattached formations in the pulp-chamber, known as pulp nodules. Any disturbance or irritation of the end of the fibrils, as from caries, abrasion, or clasps, is conveyed to the pulp, and stimulates this abnormal growth. Large amalgam fillings, through the irritation of thermal changes, contribute to this result. If the irritation is caused by decay, abrasion, or erosion the point of the pulp nearest the site of irritation will be earliest stimulated, narrowing down the size of the pulp-chamber on that side, or forming pulp nodules. This growth is built up more rapidly than normal dentine, and is also softer, wearing down more easily in abrasion. The tubes are irregular, not parallel, and often at right angles to each other. This protective growth is found in a majority of temporary teeth, when they are much decayed or abraded and are retained up to or past their normal period of life, but no modules or other unattached growths have been found. In many teeth the pulp is exhausted by long efforts in producing this growth, and dying, leaves an unoccupied space which in time causes alveolar abscesses, but at no time is the effort prolonged to a complete dentification of the pulp-chamber.

The illustrations showed a great variety of forms of abrasion and caries, with deposits of protective dentine, in both permanent and deciduous teeth, protecting the pulp in some cases, and narrowing the pulp chamber in others; also a nodule having vessels and osteo-dentine full of fibrils; another of ivory, containing an encysted bullet. In another the walls of the pulp-chamber had worn away to an angle of 45° and into the neck of the tooth, but the deposit had kept ahead of the wearing. The pulp finally died, forming an alveolar abscess. In another illustration decay at the gums and approximal surface had been followed by recession of the pulp and the formation of protective dentine until the pulp was almost cut off. This was in a deciduous tooth from a girl of thirteen years old.

Another showed an extensive deposit of abnormal dentine—not exostosis-tissue—on a wisdom tooth.

The last illustration was of a cross section of the roots of a molar cemented together with cementum all around, showing the difference between abnormal dentine and cementum.

The discussion was opened by Dr. Sudduth. He spoke of the delight it must afford every one who had taken any interest in histology to have such an exhibition as this placed before the general practitioner in a way that every one can see and understand without necessarily being an expert microscopist. It needed only eyes and ears for such an exhibition to be appreciated by any one. It should be understood that secondary dentine is developed by odontoblasts, which, especially designed for that purpose, remain quiescent on the surface of the pulp until stimulated into functional activity in the deposition of dentine, whether physiological, as in the first place, or pathological when demanded for protection from irritation, or to repair losses whenever there is interference with normal structure, or pulp irritation. The demand for secondary dentine simulates these odontoblasts to renewed functional activity. A line of discoloration precedes the cavity of decay, but the exact character of the pathological change has not yet been demonstrated; even the very exact work of Miller, of Berlin, has not yet reached this result. The field is so minute that it is almost impossible to get exact chemical results.

Dr. J. Howard Mummery next made an exhibit of photomicrographs of the minute anatomy of the tooth, describing his apparatus and his mode of bringing out the colors, etc.

Dr. Taft pronounced the combined exhibits the richest feast ever enjoyed by a dental association; that all must feel that they would be greatly profited by it.

Dr. Atkinson agreed with Dr. Taft as to the excellence of the presentment, but to truly utilize these things it would be necessary to go a little deeper than was indicated in the papers. Some histological nonsense had been repeated, and there was also shown too much lack of liberality of sentiment on the part of one worker for another. It takes a vast amount of research to entitle any one to pronounce an opinion worthy of universal respect. But we should have respect for small things, and not hurt the feelings of the youngest investigator; he might be on the very threshold of truth, and we should be wary lest we quench the light from above. All can not see with the same eyes, and very different interpretations can be put upon the same thing. We should not seek to deal with causes; not ask *why* but *how?* We must go very slow, and hold fast what we have got. He said that etiology was the great load and incubus which kept him from giving the last and best inspiration given to him. Would to God that we could get rid of the idea that we do it ourselves, and be satisfied to make known the truth as revealed to us step by step, till we become Masters in Israel, revealing the truth.

Dr. Sudduth wished to add a word about the tendency of protoplasm to assume the spheroidal form. Before cells are compressed they are all circular; the form of the cell has nothing to do with its function; the presence of fibrils does not depend on form, though Dr. Andrews laid great stress on the shape of the fibril cells. The illustrations were the most admirable demonstrations of microscopic work, and the profession should congratulate itself on having men in their ranks able to accomplish such work and willing to devote to it the time and labor necessary.

There may be many different interpretations put upon what looks alike to all in the photographs, and each one has the right to his own interpretation of what is shown us. The photographs bring out much that could not be seen even through the microscope. Some cells appear square, others pear-shaped, some are attached, others are detached, but it is a mistake to attribute any importance to the form; forms may vary, but this function remains the same. There should be no differential diagnosis from the form of the cell. He has given us improved methods of cutting, so that we may get to the living tissues before pathological changes occur. He has gone one step further in the study of osteoblasts. Osmic acid is the best preservative. A tooth extracted and preserved while warm and still bleeding from the pulp in osmic acid and alcohol is as nearly in its normal condition as we can hope to get. We will find some cells square, some triangular, some spindle-shaped, some pear-shaped, some curved. And from all points on the surface, passing alongside or through; delicate fibrils project—one, two, or half a dozen from a cell. One of Dr. Andrews' specimens showed three very distinctly. We should lay stress on function, not on form.

(After some announcements the section adjourned.)

*(To be Continued.)*

